# Cognitive decline in a patient with anti-glutamic acid decarboxylase autoimmunity; case report

**DOI:** 10.1186/1471-2377-11-156

**Published:** 2011-12-21

**Authors:** Masahito Takagi, Hiroshi Yamasaki, Keiko Endo, Tetsuya Yamada, Keizo Kaneko, Yoshitomo Oka, Etsuro Mori

**Affiliations:** 1 Department of Behavioral Neurology and Cognitive Neuroscience, Tohoku University Graduate School of Medicine, Sendai, Miyagi, 980-8574, Japan; 2 Division of Molecular Metabolism and Diabetes, Tohoku University Graduate School of Medicine, Sendai, Miyagi, 980-8574, Japan

**Keywords:** anti-glutamic acid decarboxylase antibodies, stiff person syndrome, cognitive decline, frontal dysfunction, working memory

## Abstract

**Background:**

Glutamic acid decarboxylase (GAD) is the rate-limiting enzyme for producing γ-aminobutyric acid, and it has been suggested that antibodies against GAD play a role in neurological conditions and type 1 diabetes. However, it is not known whether dementia appears as the sole neurological manifestation associated with anti-GAD antibodies in the central nervous system.

**Case presentation:**

We describe the clinical, neuropsychological, and neuroradiological findings of a 73-year-old female with cognitive dysfunction and type 1A diabetes. Observation and neuropsychological studies revealed linguistic problems, short-term memory disturbance, and frontal dysfunction. MRI showed no significant lesion except for confluent small T2-hyperintensity areas localized in the left basal ganglia. ^18^F-fluorodeoxy glucose-positron emission tomography (FDG-PET) and ^123^I-N-isopropyl-*p*-iodoamphetamine-single photon emission computed tomography (IMP-SPECT) studies showed bifrontal hypometabolism and hypoperfusion. Immunomodulating therapy with intravenous high-dose immunoglobulin resulted in no remission of the cognitive symptoms.

**Conclusions:**

Cognitive dysfunction may develop as an isolated neurological manifestation in association with type 1A diabetes and anti-GAD autoimmunity. A systematic study with extensive neuropsychological assessment is indicated in patients with type 1 diabetes and anti-GAD autoimmunity.

## Background

Glutamic acid decarboxylase (GAD) is the biosynthesizing enzyme of the neurotransmitter γ-aminobutyric acid (GABA). Antibodies against GAD cause neurological syndromes[1], including stiff person syndrome (SPS)[2], cerebellar ataxia[3], and limbic encephalitis[4] as well as type 1 diabetes[5]. Behavioral and cognitive problems may be associated with SPS[6], limbic encephalitis[7], or cerebellar ataxia, and some of the psychiatric symptoms that have been reported in SPS[8] are considered to be related to dysfunction of the GABAergic system. However, it is not known whether dementia appears as the sole neurological manifestation associated with anti-GAD antibodies in the central nervous system. We report here a patient with GAD autoimmunity and type 1A diabetes who developed cognitive impairment without known anti-GAD-related neurological conditions.

## Case Presentation

A 73-year-old, right-handed, high school-educated Japanese housewife developed polydipsia, polyuria, progressive weight loss, and increasing fatigue in the summer of 2008. A diagnosis of type 1 diabetes was made, and the patient was admitted to our hospital in February 2009 to control her diabetes. The attending physician and nurses in the ward noticed that she had difficulty mastering insulin self-injection, and she was referred to us for evaluation of possible dementia. She lived independently, and she and her family had not noticed memory problems in her daily life. She had no history of cigarette smoking, alcohol abuse, or neurological/psychiatric illness. A detailed review of the family history was unremarkable for neurologic/psychiatric illness.

On examination, the patient was oriented to place, but not to time. There were no signs of mood disorders, psychiatric illness, or changes in personality or social conduct. The neurological examination was unremarkable; the only faint abnormality we detected was an irregular saccadic eye movement on lateral gaze with difficulty maintaining rightward gaze.

The results of routine laboratory tests were all within normal limits except for mild hyperglycemia (serum glucose 128 mg/dl, HbA1c 7.2%). Her thyroid function was normal, and her serum levels of vitamin B_1 _and B_12 _were also normal. The serological studies indicated high titers of anti-GAD (2865.2 U/ml), anti-insulinoma associated protein (IA)-2 (45.1 U/ml), anti-thyroid peroxidase (14.5 U/ml), and anti-thyroglobulin (67.8 U/ml) antibodies. Her cerebrospinal fluid (CSF) was negative for hypercellularity, oligoclonal bands, or myelin basic protein. Her CSF was positive for anti-GAD antibodies (60.1 U/ml). The antibody specificity index (ASI = [anti-GAD_CSF_/IgG_CSF_]/[anti-GADserum/IgGserum], which measures the intrathecal synthesis of anti-GAD antibodies[9,10]) was 3.16, while the IgG index was 0.53.

**Figure 1:**
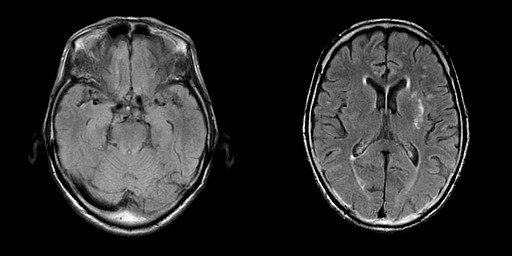
**The brain MRI findings**. The axial fluid-attenuated inversion recovery (FLAIR) images showed a small hyperintense lesion in the left putamen. Neither generalized nor focal cortical atrophy suggestive of Alzheimer disease or other degenerative dementias was noted.

**Figure 2:**
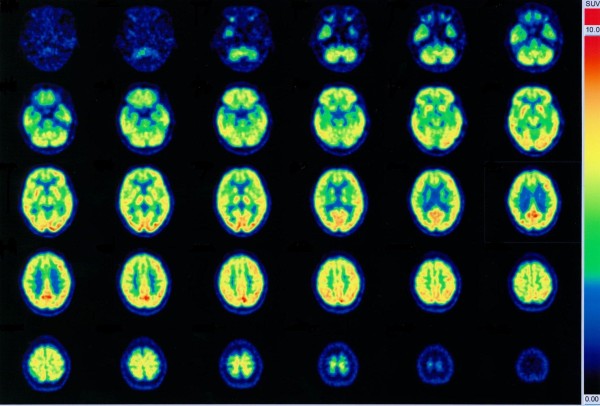
**The FDG PET scans of the patient**. Bilateral frontal lobe hypometabolism was noted.

The thoracic, abdominal, and pelvic CT scans showed no evidence of malignancy. An MRI of the head did not demonstrate any abnormalities other than a small and questionable lesion showing T2-hyperintensity not associated with T1-hypointensity in the left putamen (Figure 1). Specifically, there was no evidence of atrophy of the medial temporal lobes. The functional neuroimaging, ^18^F-fluorodeoxy glucose-positron emission tomography (FDG-PET) indicated bifrontal cortical hypometabolism (Figure 2) and ^123^I-N-isopropyl-*p*-iodoamphetamine-single photon emission computed tomography (IMP-SPECT) showd concomitant hypoperfusion. Carotid Doppler ultrasonography showed mild atherosclerotic change with a maximum intima-media thickening of 2.0 mm. The EEG showed mild general slowing and bilateral temporal delta-range activity.

Table 1 summarizes the results of the neuropsychological tests. The patient' speech was fluent, and her articulation and prosody were normal. There were few literal and semantic paraphasias. However, she had apparent language problems characterized by defective auditory comprehension and defective repetition. Her score on the Japanese version of the Western Aphasia Battery AQ was 78.4. Her language problems were also apparent when comparing her WAIS-III verbal IQ to her relatively preserved performance IQ. Her executive functions were also defective; the verbal fluency, Trail making-B, and WAIS III working memory sub-items indicated low performance, while her processing speed was preserved. The memory tests revealed that the patient was mildly amnestic. Her recognition memory was relatively preserved.

**Table 1:** The Patient's Neuropsychological Profile


***General Intelligence***	
**Wechsler Adult Intelligence Scale-3rd edition**	
Full Scale IQ	70
Verbal IQ	66
Performance IQ	80
Verbal Comprehension	66
Perceptual Organization	77
Working Memory	67
Processing Speed	102
***Attention***	
**Digit Span**	
Forward	3
Backward	3
**Tapping Span**	
Forward	4
Backward	2
**Wechsler Memory Scale-Revised**	
Attention/Concentration (index)	53
***Language***	
**Western Aphasia Battery**	
Aphasia Quotient	78.4
***Memory***	
**Wechsler Memory Scale-Revised**	
General Memory	64
Delayed Recall	63
**Rey Osterrieth Complex Figure Test**	
Immediate Recall	10.5/30
Recognition	19/24
**Rey Auditory Verbal Learning Test**	
Total recall during trial 1-5	23/75
Recognition	11/15
***Visuoperceptual Ability***	
**Cube copy**	perfect
**Rey Osterrieth Complex Figure Test**	
Copy	36/36
***Executive Function***	
**Verbal Fluency Test**	
Category (Animal)	11
Phoneme (fu, a, ni)	3,5,3
**Trail Making part A**	62 sec
**Trail Making part B**	249 sec
***Nonverbal Reasoning***	
**Raven Colored Progressive Matrices Test**	24/36

A five-day course of high-dose (0.4 g/kg/day) intravenous immunoglobulin (IVIg) was implemented after written informed consent was obtained. However, the symptoms, neuropsychological profile, and insulin dependence remained unchanged immediately after the treatment. Her anti-GAD antibody titers also remained high, both in her serum (2832.5 U/ml) and CSF (75.4 U/ml). No further treatment was implemented due to her withdrawal of consent. The status of her diabetes has been stable for one and a half years. No progression of neurological impairment has been demonstrated in daily life, on the neuropsychological tests, and on neuroimagings during this period.

## Discussion

We have described here a type 1 diabetic, anti-GAD antibody-positive patient who presented with cognitive impairment characterized by language disturbance, verbal short-term (working) memory disturbance, executive dysfunction, and mild amnesia with spared recognition memory as an isolated symptom. Although behavioral and cognitive impairments may also accompany anti-GAD-related neurological disorders, including cerebellar ataxia and limbic encephalitis, no signs of these disorders were detected in the patient. The cognitive impairment is of features of frontal lobe involvement, which was not improved significantly after a single course of high-dose IVIg treatment, although no further progression was noted thereafter over 18 months.

The non-progressive time course of the cognitive decline is not supportive of neurodegenerative diseases, and the features of the cognitive impairment and of neuroimaging findings are not compatible with Alzheimer diseases, the commonest cause of dementia in the patient's age, although it can not be completely ruled out. The small lesion restricted in the left putamen shown by the MRI is unlikely to be causative of the cognitive impairment, as the putamen is not a strategic site interrupting frontal-subcortical circuits for behavior and cognition [11].

Recent studies have demonstrated that cognitive decline is associated with type 1 diabetes. A meta-analysis[12] has indicated that type 1 diabetes-related cognitive dysfunction is characterized by decreased mental speed and mental flexibility with spared learning and memory, which is consistent with the cognitive impairment of our patient. Although the underlying mechanisms remain to be elucidated, cognitive impairment related to frontal lobe dysfunction may occur in patients with type 1 diabetes. Increased risk for cerebrovascular diseases in diabetic patients is generally accepted to be attributable to the cognitive decline of features of frontal involvement in those patients. Anti-GAD immunity has not been considered to be directly causative of cognitive decline in type 1 diabetics. Meanwhile, our patient was positive for anti-GAD antibody in CSF, which made us infer some possible autoimmune process occurred in cerebrum resulting in cognitive dysfunction. A recent study has revealed that high anti-GAD antibody levels (more than 2000 U/ml) are associated with a spectrum of neurological syndrome[1], and that the intrathecal synthesis of anti-GAD antibodies plays an important role in the development of neurological disorders. In our patient, the serum anti-GAD antibody level was as high as that of SPS patients, and the ASI for anti-GAD antibodies exceeded the IgG index and the normal range[9], which supports anti-GAD autoimmunity as the potential cause of the cerebral involvement.

Immunomodulating therapies are reportedly effective for anti-GAD autoimmunity -associated neurological manifestations [4,7,13,14]. A single case report described a patient with Stiff-person plus syndrome[15] in whom anxious depression was improved after corticosteroid treatment. Although a few case reports showed that neurological manifestations are along with decrease of anti-GAD antibody titer, no correlation was noted between decrease of anti-GAD antibody titers and the magnitude of clinical response in a controlled study, in which a three-month course of high-dose IVIg demonstrated benefits in SPS patients [16]. Therefore, it is difficult to discuss the relationship between the failure of serological and clinical response after high-dose IVIg in our patient. Although an optimal treatment for GAD anti-immunity has not been established, high-dose IVIg seemed to be most promising at least for SPS. Monthly repetitive administration of 2 g/kg immunoglobulin divided into two daily doses (i.e., 1 mg/day × 2) has been originally recommended, and the response reportedly appears after repeated dosages of several months. We followed the recommendation for the treatment of neuromuscular disease in our country, the same dosage divided into five daily doses (i.e., 0.4 mg/day × 5), to minimize infusion reactions. As we could give only one course of IVIg because of the patient's refusal, we cannot conclude whether the treatment is ineffective or insufficient.

## Conclusion

Our patient suggests that dementia occurs as a potential isolated neurological manifestation of anti-GAD autoimmunity, and that anti-GAD autoimmunity is a possible cause of cognitive decline in patients with type 1 diabetes. As cognitive decline is a common, rather nondescript symptom, a systematic study with extensive neuropsychological assessment would be indicated in patients with anti-GAD autoimmunity and type 1 diabetes.

## Consent

Written informed consent was obtained from the patient for publication of this case report and accompanying images. A copy of the written consent is available for review by the Editor in Chief of this journal.

## List of abbreviations

ASI: antibody specificity index; CSF: cerebrospinal fluid; FDG-PET: ^18^F-fluorodeoxy glucose-positron emission tomography; GABA:γ-aminobutyric acid; GAD: Glutamic acid decarboxylase; IMP-SPECT: ^123^I-N-isopropyl-*p*-iodoamphetamine-single photon emission computed tomography; IVIg: intravenous immunoglobulin; SPS: stiff person syndrome.

## Competing interests

The authors declare that they have no competing interests.

## Authors' contributions

MT drafted the first manuscript and made a contribution to acquisition and interpretation of data. HY is a treating neurologist of the patient, and made a contribution to acquisition and interpretation of data. KE is a speech therapist of the patient, and made a contribution to acquisition and interpretation of data. KK and TY are treating physicians of the patient, and made a contribution to data acquisition and literature search.

YO and EM supervised this study, participated in its design and coordination, and revised the manuscript that led to the final approval of the current submission. All authors read and approved the final manuscript.

## Pre-publication history

The pre-publication history for this paper can be accessed here:

http://www.biomedcentral.com/1471-2377/11/156/prepub
